# Involuntary Cognitions in Everyday Life: Exploration of Type, Quality, Content, and Function

**DOI:** 10.3389/fpsyt.2015.00007

**Published:** 2015-02-02

**Authors:** Julie Krans, June de Bree, Michelle L. Moulds

**Affiliations:** ^1^Faculty of Psychology and Educational Sciences, Behavior, Health and Psychopathology Research Group, University of Leuven, Leuven, Belgium; ^2^Department of Clinical Psychology, Radboud University Nijmegen, Nijmegen, Netherlands; ^3^School of Psychology, University of New South Wales, Sydney, NSW, Australia

**Keywords:** intrusions, mental imagery, intrusive cognitions, involuntary memory, psychopathology

## Abstract

Psychological research into spontaneous or intrusive cognitions has typically focused on cognitions in one predefined domain, such as obsessional thoughts in OCD, intrusive memories in posttraumatic stress disorder and depression, or involuntary autobiographical memories and daydreaming in everyday life. Such studies have resulted in a wealth of knowledge about these specific cognitions. However, by focusing on a predefined type of cognition, other subtypes of cognition that may co-occur can be missed. In this exploratory study, we aimed to assess involuntary cognitions in everyday life without a pre-determined focus on any specific subtype of cognition. Seventy unselected undergraduate student participants were administered a questionnaire that assessed the presence of any involuntary cognitions in the past month, their quality, type, content, and potential function. In addition, participants provided self-descriptions and completed measures of psychopathology. Content analyses showed that involuntary cognitions were common, predominantly visual in nature, emotional, often about social relationships, and often related to a hypothetical function of emotional processing. About two-thirds of the cognitions that participants reported were memories. Non-memories included daydreams, imaginary worst case scenarios, imaginary future events, hypothetical reconstructions, and ruminations. Memories and non-memories were strikingly similar in their subjective experience of content and emotionality. Negative (but not positive) self-descriptions were associated with negative involuntary cognitions and psychopathology, suggesting a link between involuntary cognitions and the self. Overall, the findings suggest that people experience a wide variety of subtypes of involuntary cognitions in everyday life. Moreover, the specific subtype of involuntary cognition appears to be less important than its valence or content, at least to the subjective experience of the individual.

## Introduction

The field of psychology is characterized by a historical interest in the ways of the mind that appear to operate beyond voluntary control. For example, Sigmund Freud inspired an interest in subconscious processes that operate outside of our volition. Within the domain of psychopathology there has been a marked interest in involuntary phenomena such as psychotic hallucinations, obsessions, and traumatic flashbacks. In cognitive psychology, a growing body of research has investigated the occurrence of involuntary memories experienced in everyday life by individuals in the general population. These interests in involuntary processes across the clinical and cognitive psychology literatures have resulted in numerous scientific publications on specific forms of involuntary cognitions; i.e., thoughts, memories, or images that come into awareness in the absence of an intention of conjuring them. In this study, we aimed to explore involuntary cognitions in everyday life using a bottom-up approach that is free from the restrictions of any theoretical framework or an *a priori* restrictive focus on any one subtype of involuntary cognition.

Several theorists and researchers have sought to describe and explore features of different forms of involuntary cognitions. Some studies sought to compare involuntary cognitions between clinical and non-clinical populations, whereas others assessed their occurrence in everyday life. Below we describe the main observations that have emerged from this literature. Throughout this literature, many different terms have been used to describe the type of cognition that was studied (e.g., obsessions, intrusive thoughts, and involuntary memories), although a definition of the adopted terms is not always provided. When reviewing the literature, we will describe the research findings using the original terminology adopted by the authors, and provide their definitions where available. In our own writing, we use the term “cognition” as an umbrella term for memories, thoughts, and images. “Memories” refer to representations in autobiographical or semantic memory, “thoughts” refer to verbal thoughts, and “images” refer to mental images from sensory modalities, most commonly visual. Thoughts and images may or may not be part of a memory representation.

### Involuntary autobiographical memories

Involuntary negative autobiographical memories (often referred to in the literature as “intrusive memories” owing to the fact that they are distressing and intrude into consciousness unbidden) represent another type of involuntary cognition that has been investigated across a range of clinical disorders (1). Involuntary memories of negative events have been characterized and studied most extensively in posttraumatic stress disorder (PTSD), a clinical condition in which the reliving of one’s traumatic experience via the experience of recurrent and distressing memories is a key diagnostic criterion. Beyond PTSD, a growing evidence-base has documented the presence of involuntary negative memories, as well as other mental images, in a range of disorders, including panic disorder with agoraphobia (2), social phobia (3), bipolar disorder (4), grief (5), and depression (6). However, although involuntary memories of negative events are reported by clinical samples, it is noteworthy that such memories are also reported by healthy individuals. For example, Newby and Moulds (6) found that there was no significant difference between the proportions of currently depressed, formerly depressed, and never-depressed individuals who had experienced an involuntary memory of a negative event in the previous week. Interestingly, the phenomenological experience of involuntary negative autobiographical memories (e.g., here and now quality, reliving) was strikingly similar across the three groups; however, currently depressed participants reported that their memories were more vivid, distressing, caused greater interference, and were associated with more sadness, helplessness, and anger.

Turning to studies on this topic outside of the clinical literature, Brewin et al. (7) assessed intrusive thoughts and intrusive autobiographical memories in a non-clinical sample. They defined intrusive thoughts as “spontaneous, repetitive thoughts” that interrupted ongoing activities and were hard to control, including images, impulses, or ideas. Intrusive memories were defined as memories of a specific event that interrupted daily activities and were hard to control. Participants were asked to report five intrusive memories and five intrusive thoughts and to rate their frequency in the past 2 weeks, as well as their level of (un)pleasantness. After controlling for the order of recall (i.e., memories first or thoughts first), the only difference that emerged was that thoughts tended to intrude more often than memories.

Studies conducted outside of the laboratory have also investigated involuntary autobiographical memories in everyday life. For example, in an elaborate diary study, Berntsen (8) asked 14 participants to record 50 involuntary memories with a maximum of 2 memories per day. Participants answered a series of questions about each memory. In addition to the diary, participants were asked to estimate how many involuntary memories they experienced on an average day. Participants reported that they experienced more than two involuntary memories per day, with five or six involuntary memories per day being the most typical estimate (range 3–20). Almost all memories were cued by triggers that shared salient features with the memory, with activities, objects, and people being the most common triggers. The valence of the memories was often congruent with participants’ mood state at the time of recall [see also Ref. (9)]. Most of the memories were of unusual events, such as traveling abroad, parties, and romantic encounters, and memories were more often rated as positive rather than negative [see also Ref. (10)]. Participants reported an increase in positive mood as a result of recalling positive memories, and an increase in negative mood from recalling negative memories.

In another study, using Galton’s “memory walk” as well as a word-cue task, Berntsen and Hall (11) found that involuntary autobiographical memories, compared to voluntary memories, were more often of specific events as opposed to general events, and evoked more intense physical and emotional reactions. Using diary monitoring, Schlagman and Kvavilashvili (12) found that participants experienced an average of 17 involuntary autobiographical memories over a period of 1 week.

### Involuntary semantic “memories”

In other studies, researchers have examined instances in which semantic information comes to mind spontaneously. Kvavilashvili and Mandler (13) referred to these instances as “involuntary semantic memories,” and defined them as information without context and not related to the self, such as words, images, or songs. In a study with 205 psychology students, Kvavilashvili and Mandler (13; Study 3) administered a questionnaire that asked participants whether they had experienced involuntary semantic memories or “mind-popping.” Of the participants, 84% indicated that they had at any time in their life experienced involuntary semantic memories. On average, participants estimated that these memories occurred between 1–2 and 3–4 times per week. The most frequent type of memory was a familiar tune popping into mind (indicated by 80% of participants).

In a subsequent study (13; Study 4), the authors compared involuntary semantic memories to involuntary autobiographical memories obtained from 50 psychology students. Participants were asked to record their involuntary autobiographical memories in a diary for 1 week, and their involuntary semantic “memories” in a diary during another week (in counterbalanced order). Overall, 205 involuntary autobiographical memories and 74 involuntary semantic memories were reported, which was a statistically significant difference in frequency. Similar to the earlier study, involuntary semantic memories were of words (61%), songs (27%), and images (12%). The authors did not report on the content of the autobiographical memories. Both memory types were associated with relatively low levels of cognitive demand or concentration. Eighty percent of the involuntary autobiographical memories were associated with identifiable triggers, whereas this was only the case for 37% of the involuntary semantic memories, which was a statistically significant difference.

### Involuntary thoughts and images

In an early paper on this topic, Rachman and De Silva (14) reported the findings of two studies in which they assessed obsessions in a non-clinical and a clinical sample of “obsessional patients”; i.e., individuals who sought clinical help for their obsessions. Obsessions were defined as “repetitive, unwanted, intrusive thoughts of internal origin” (p. 233). In the first study, 99 out of 124 non-clinical participants reported experiencing obsessional thoughts, which they generally found easy to dismiss. Participants varied in the extent to which they rated their obsessions as tolerable. In the second study, information about obsessions was obtained from clinical and non-clinical participants. Blind raters with clinical experience were presented with the obsessions and asked to judge whether each one was reported by a clinical or non-clinical participant. The raters were fairly accurate in identifying the obsessions from the non-clinical participants as belonging to the non-clinical group. However, for the obsessions reported by clinical participants, raters did not do well in identifying these obsessions as belonging to the clinical group. This indicated that the content was quite similar in both groups. The obsessions reported by participants in the clinical group were experienced as longer, more frequent, more intense, and less acceptable and dismissible than the obsessions reported by participants in the non-clinical group. A later study by Salkovskis and Harrison (15) replicated these results and further reported that participants’ discomfort with experiencing the obsessions was not related to obsession type (i.e., “thoughts” or “impulses”), but by the ease with which they could be dismissed.

Clark and De Silva (16) asked non-clinical participants to rate a list of six depressive and six anxious cognitions (i.e., thoughts and images) on several dimensions. On average, participants reported that these cognitions occurred between twice a month and once a week. Anxious cognitions were rated as more emotionally intense than depressive cognitions, and the perceived controllability of both anxious and depressive cognitions predicted their frequency. In turn, frequency and emotional intensity (and disapproval for anxious cognitions only) predicted participants’ ratings of controllability.

Beyond studies of involuntary memories and involuntary thoughts, researchers have investigated other intrusive cognitive phenomena and processes, including mind wandering (17, 18) and involuntary images of future events [“flashforwards”; (19)].

### Present study: Involuntary cognitions

Taken together, the studies outlined above provide valuable insights into an array of specific subtypes of involuntary cognitions that have been indexed across studies in the clinical and non-clinical literatures. Although investigating different types of involuntary cognition, a common feature of these studies is that the researchers identified *a priori* the subtype of involuntary cognition that was to be assessed. This may be an important limitation of the literature, because it may well be that people experience a range of different types of involuntary cognition. Focusing on one or two pre-determined subtypes of involuntary cognitions precludes the collection of data on other subtypes, and limits examinations of their relative importance in everyday life. That is, when wanting to determine the content of a fruit basket, one can decide to examine only cherries, or apples, or bananas. However, by doing so, the full content of the basket will never be known. In light of the abovementioned limitations, in this study we adopted a bottom-up approach as a means by which to take a first step into exploring the full scope of involuntary cognitions in everyday life as they are experienced by an unselected sample of individuals.

Given that involuntary cognitions are accepted as a transdiagnostic occurrence across many disorders [e.g., Ref. (20)] we also explored the link between different types of involuntary cognitions that would emerge from our procedure and psychopathology. The link between involuntary cognitions and psychopathology has been described in the Self-Memory System Model [SMS; (21)]. According to this model, the content of involuntary images should be related to psychopathology through the individual’s self-images. In order to explore this link, self-descriptions were also obtained.

## Materials and Methods

### Participants

Seventy participants (58 females) were recruited from the student population of the Radboud University Nijmegen. Of these, 68 were undergraduate students, one was a graduate student, and one recently dropped out of their course. Participants received a €10 gift voucher for their participation, and ranged in age from 18 to 37 (*M* = 21.71, *SD* = 3.65).

### Involuntary cognitions questionnaire

This self-report questionnaire was developed for the purpose of this study and the structure was based on earlier intrusive memory interviews (22–24). First, participants read an introduction about involuntary cognitions, which read:

Many people have the experience that in some moments a certain image, a certain thought, or a certain memory comes to mind, without them deliberately thinking about this. These can be positive as well as negative experiences. Someone who is in love, for example, sees the face of his or her loved one in their mind time and time again. Someone who just experienced a car accident can keep seeing images of this experience in their mind. It is also possible that someone frequently sees a certain image in mind without exactly knowing where this is coming from. For example, someone can see an image of themselves completely alone, or trapped, or covered in germs. What is important here is that it is about images, thoughts, or memories that come in your mind spontaneously without you deliberately thinking about it.

Participants were then asked whether they had experienced any such spontaneous images, thoughts, or memories within the last month. If they had, participants were then asked to provide a detailed description of each image/thought/memory, which included its content (e.g., location, people, event, and time), personal meaning, whether it was a memory of an actual event that they had experienced (yes/no), which (if any) emotions were associated with the involuntary cognition (fear, anger, sadness, disgust, happiness, or other), and how strongly they experienced any such emotions (1 = *not at all*, 7 = *very strongly*). After completing these questions, participants were asked whether they had experienced another spontaneous image, thought, or memory within the past month. If so, the questions were repeated for this involuntary cognition. Participants were queried about a maximum of three involuntary cognitions.

### Coding scheme

The descriptions of the involuntary cognitions on the involuntary cognitions questionnaire (ICQ) were coded independently by two raters (Julie Krans and June de Bree) on the characteristics of quality, type, content, and exploratory, possible function. Disagreements were resolved based on consensus after discussion.

#### Quality

For quality, involuntary cognitions were classified according to their dominant quality (i.e., visual, verbal, emotional, or bodily sensation). These categories were based on earlier studies on the qualities of intrusive memories (22–24). Inter-rater agreement for quality was *K* = 0.472.

#### Type

For type, participants’ response to the question about whether the involuntary cognition was a memory was used to identify spontaneous cognitions as either memories or non-memories. We initially intended to code non-memory cognitions according to existing categories (25), but this resulted in a high number of unclassifiable cognitions. Accordingly, we classified non-memory involuntary cognitions into the following categories, on the basis of the coders’ ratings (i.e., Julie Krans and June de Bree; *K* = 0.666): daydream, worst case scenario, future event, hypothetical reconstruction (descriptions that fill gaps in the information that the participant has about a certain event), or rumination (recurring and extended verbal dwellings on a certain topic).

#### Content

The content of the involuntary cognitions were coded into categories based on the classification systems developed to study self-defining memories (25, 26), on the basis that these categories were a good fit for the involuntary cognitions reported by participants (*K* = 0.714). These categories were as follows: relationships (emphasis is on a particular interpersonal relationship), life-threatening events (plausible or actual death or threatened physical well-being of oneself or someone else), recreation/exploration (recreation, play or exploration or obstruction of these), achievement/mastery (the effortful striving toward or achievement of own, in-group, or significant others’ physical, material, social, or spiritual goals), guilt/shame (issues of wrong or right, moral, and ethical decisions), and not classifiable (not belonging to any of these categories).

#### Function

The function categories were the most explorative of all categories. Although the literature of function of autobiographical memory is rather substantive [e.g., Ref. (27)], to our knowledge, no empirical research has been published on functions of involuntary recall. According to a functional analysis of involuntary memories (28), the theoretical literature has suggested several possible “functions.” Therefore, we borrowed from the autobiographical memory literature and the trauma literature to develop our “function” categories.

##### Emotional processing

First, according to the Self-Memory System of autobiographical memory (21), involuntary memories persist when there is a discrepancy between an individual’s current state and desired state. The content of the involuntary memory provides information about the nature of this discrepancy. In that sense, involuntary memories “signal” that something is wrong and action needs to be taken to reduce a certain unwanted discrepancy in the psychological self. The dual representation theory of PTSD (29) suggests that in trauma, involuntary memories provide the information that is necessary to emotionally process the traumatic event. That is, a traumatic event often induces a discrepancy in the self (not dissimilar to the discrepancy put forward by the Self-Memory System), which can be dealt with by integrating all available information about the event with other knowledge in autobiographical memory. The theory proposes that highly emotional sensory information is by definition stored in involuntary memories, hence these memories provide the necessary information to successfully emotionally process the event. Taken together, these two models appear to converge on the idea that involuntary memories contain information that aids in the emotional processing of an emotional experience. Here, this category included involuntary cognitions that emphasized an event that still required closure, such as loss or holding a continuing grudge against someone about a wrong-doing in the past.

##### Maintaining social relations

In the autobiographical memory literature it has been suggested that recalling (and sharing) autobiographical memories about significant others is involved in the maintenance of social relationships (30). It is possible that involuntary memories or other cognitions about significant others may be helpful for the same reason. That is, recurring involuntary cognitions about friends, family, or other social relations may stimulate the social relation by keeping the memory active. Therefore, involuntary cognitions that emphasized a specific relationship with a significant other were assigned to this category.

##### Mood repair

In the emotional memory and depression literature, it has been found that individuals can improve a sad mood state by recalling positive autobiographical memories [e.g., Ref. (31–33)]. Positive images especially are expected to have an effect on mood (34). Hence, it is possible that spontaneous occurrences of positive images can have the same effect. Accordingly, involuntary cognitions that emphasized positive events or feelings were assigned to this category.

##### Preparation

Thinking about or imagining future events has been implicated in the preparation for and likelihood of actual behavior in the future (34, 35). Similarly, involuntary cognitions about upcoming events may provide an opportunity for preparation and increase likelihood of future actions. Involuntary cognitions that predominantly emphasized upcoming events were assigned to this category.

##### Warning signal

This may be the most concrete proposed function of involuntary memories, at least in the trauma literature. According to the warning signal hypothesis [e.g., Ref. (22)], involuntary memories of negative events come to mind in situations that resemble the physical characteristics of this earlier negative experience. In this way, the involuntary cognition functions as a “warning signal” for potential danger in the present situation. Involuntary cognitions of negative events that were triggered by (a subjective sense of) similarity with the current situation were assigned to this category. We note one difference between Ehlers et al. (22) warning signal hypothesis and our conceptualization of this function in the current study: the involuntary cognitions reported by participants in this study were not restricted to actual memories of a traumatic event.

### Self-descriptions

Self-descriptions were assessed with the Twenty-Statements Test [TST; (36, 37)]. The TST assesses individual self-descriptions as conceptualized in the SMS model by asking participants to complete 20 sentences starting with “I am…” (37). These descriptions were subsequently coded as positive (e.g., “I am smart”), negative (“I am ugly”), and neutral (“I am curious”) self-descriptions (*K* = 0.785).

### Psychopathology

The Dutch version of the Symptom Checklist 90 [SCL-90; (38)] is a 90-item self-report questionnaire that assesses perceived current physical and mental complaints aimed to reflect a general level of psychopathology. It is one of the most widely used screening questionnaires in Dutch mental health facilities. The total score is an indicator of general physical/mental dysfunction or “psychoneuroticism.” In addition, the following subscales can be identified: Somatization, Obsessive compulsive, Interpersonal sensitivity, Depression, Anxiety, Hostility, Phobic anxiety, Paranoid ideation, Psychoticism, and additional items. In a Dutch sample of individuals who contacted a mental health facility for the first time the internal consistency was α = 0.97 (39).

### Procedure

Participants were tested individually in a testing cubicle in the lab of the Behavioral Science Institute. They completed a demographics questionnaire, the ICQ, followed by several measures that are not relevant to the present study (assessing depression, anxiety, goals, and self-esteem) and the SCL-90. For more information on all measures that were included in the study please contact the corresponding author. Afterwards participants were debriefed, thanked, and paid. The entire session was usually completed within about 45 min. All questionnaires and tasks were completed on a PC using Inquisit version 2.0 (40).

## Results

### Frequency

In total 62 involuntary cognitions were reported. Of the 70 participants, 23 (32.86%) reported not having experienced any involuntary cognitions in the past month. A further 34 participants (48.57%) reported 1 involuntary cognition, whereas 11 (15.71%) and 2 (2.86%) participants reported 2 and 3 involuntary cognitions, respectively. Thus, the majority of all participants, which is 67.14% of the present sample, recalled having experienced at least one involuntary cognition in the past month.

### Associated emotions and emotional intensity

For two participants no emotion data were recorded due to a technical error. On the ICQ participants were presented with the options “fear,” “anger,” “sadness,” “disgust,” “happiness,” or “other.” For 13 involuntary cognitions, the “other” category was endorsed after which participants were asked to label the associated emotion. The “other” category emotions were labeled by the participants as “distress,” “love,” “doubt,” “shame,” and “relaxed.” The large majority of involuntary cognitions were associated with either happiness (20; 32.26%) or sadness (15; 24.19%). These two categories far outweighed all other categories. Table 1 presents the frequency with which each emotion was endorsed and the mean intensity score for each emotion.

**Table 1:** **Frequencies of endorsed emotions for all involuntary cognitions in descending order and the mean emotional intensity per emotion**.

Emotion	Frequency	Intensity	
		*M*	SD
Happiness	20	6.05	0.74
Sadness	15	5.67	0.90
Fear	6	4.83	0.98
Distress	6	5.00	0.84
Anger	3	5.33	0.58
Disgust	3	6.33	0.58
Love	3	6.00	0.00
Doubt	2	5.50	0.71
Shame	1	6.00	n.a.
Relaxation	1	6.00	n.a.

Overall, the reported involuntary cognitions were highly emotional, with average intensity ratings across emotions from 4.83 to 6.33 on a 1–7 scale. Due to low numbers of involuntary cognitions that were associated with emotions other than happy or sad, no statistical analyses were performed to test for differences in emotional intensity.

### Quality

The large majority of involuntary cognitions (82.26%, *n* = 51) were predominantly visual in nature. A further 8.06% (*n* = 5) were predominantly verbal in nature, four (6.45%) were predominantly an emotional experience, one was a bodily sensation, and one could not be coded into any of these categories.

### Type

For more than half of the involuntary cognitions (61.29%, *n* = 38), participants indicated that the content was a memory of a specific event that had occurred in their lives. Thus, the remaining 24 involuntary cognitions were not memories. These non-memories were classified into the Type categories. Table 2 presents the frequency for each category, as well as an example.

**Table 2:** **The type of non-memory involuntary cognitions with their frequency and an example for each**.

Type	Frequency
Daydream	7

Example:
*I sometimes see a person with whom I might be in love, we are in a room and he looks at me and I have the feeling that he honestly thinks that he loves me. Besides us no one else is in the room. I have several feelings with it, on the one hand it’s very beautiful to me, and on the other hand it’s not, because in theory I shouldn’t have feelings for him. Still I think it’s beautiful, when the image appears*	

Worst case scenario	5

Example:
*Last Friday I was at my parents’ house and there was no one else at home, only our 2 dogs were there. I was brushing/grooming one of them and the TV was on at the same time, where a movie was playing. Suddenly the image came to mind that my brother would not make it through the job application, that his world collapsed, including my parents’ and therefore of the entire family. There was a lot of pressure, because in times of this economic crisis as a [occupation] he would not get a new chance soon. After I had let the image come over me, a worried and jaded feeling came over me, also because it could come true. There was indeed a chance that he would not make it. Of course you take this into account, but still*	

Future event	5

Example:
*The location is [city name] and I’m there with my future friends. It’s about the art academy where I’m going in [month] it is an image instead of a memory. This means a lot to me because I really want it. I have positive thought with it. I’m happy but also a little insecure of whether I will be able to do it*	

Hypothetical reconstruction	4

Example:
*On the occasion of break-in at home. Backyard, 2 burglars, walking through the backyard to the backdoor, seeing exactly how they would have done it. It happens at night. Does not really mean anything to me, I try to ignore it. It evokes feelings of fear, doubt whether they will come back again and if it really happened this way. Desire to see the movie in real life so that I really know how it happened*	

Rumination	3

Example:
*When I’m alone then often memories come up of my relation that ended 2 weeks ago. I mostly have this when I’m alone, then I start wallowing in my head and then memories come up. Or when I hear a specific music track that is associated with a memory or that I encounter things that remind me of the 4.5 year relationship. So my ex is involved in this. And it takes place mostly at night when I’m alone in my room and don’t have distraction from other people. It evokes mixed feelings, feelings of loss but also negative feelings about everything that went wrong and how much it has hurt me. I often am furious but I can also burst into tears because it has become such a mess. And I often have to tell myself: Come on, you deserve better. But that is often easier said than done*	

### Content

Half of all involuntary cognitions were about relationships. The next most frequent content concerned life-threatening events (*n* = 15; 24.19%), followed by recreation/exploration (*n* = 8; 12.90%), achievement/mastery (*n* = 4; 6.45%), and guilt/shame involuntary cognitions (*n* = 2; 3.23%). Two involuntary cognitions were marked as not classifiable, as they did not fit into any of these categories. Table 3 presents an example of an involuntary cognition for each of the categories.

**Table 3:** **Examples of each of the Content categories**.

Content category	Example
Relationships (*n* = 31)	*There is not really a location. It is about my father whom I haven’t seen for about 14 years due to circumstances. I visualize him and me in a living room where we catch up on the last 14 years. The time when it plays is also unclear. The image means quite a lot to me. I would like to speak to him again and stuff, but it doesn’t work the way I want it to. The thoughts I have with this are tricky, because I don’t know what he is like now and how I will respond to that in turn. It evokes (again) feelings of being incomplete*
Life-threatening event (*n* = 15)	*A car came by very closely and I already saw myself lying under the car. I was alone. I felt very nervous afterwards*
Recreation/exploration (*n* = 8)	*A holiday in America with a friend who was an au pair there for a year. We are cycling in the sun, on our way to the supermarket for groceries. This is sometime during the course of a weekday, on which my friend had to work as usual. This image makes me happy, I think back to this holiday as very successful, a beautiful experience. It was also a unique experience. In relation to the weather in the Netherlands I think back to it and then I feel a little nostalgic. Especially the weather is important in this memory. Also the feeling of being happy, not to have to do things, just holiday and relaxation*
Achievement/mastery (*n* = 4)	*I think a lot about a job interview that I have in the near future. It is set in a room at the [name university]. The feelings it evokes I can perhaps best describe as ‘healthy stress’. I think about it a lot because I really want to be admitted, but that also causes mild tensions*
Guilt/shame (*n* = 2)	*Last week when I was lying in bed a shameful event came up spontaneously that was not nice at all at that moment. The same shameful feeling arose and I wanted mostly to cringe*

### Function

Of all involuntary cognitions, 22 (35.48%) were assigned to the category emotional processing, 15 (24.19%) to maintaining social relations, 12 (19.35%) to warning signals, 8 to mood repair (12.90%), and 5 (8.06%) to preparation. Table 4 provides examples for each category.

**Table 4:** **Examples for each of the function categories**.

Category	Example
Emotional processing (*n* = 22)	*Location: the apartment where I used to live with my boyfriend at the time. What happens: my sister and my boyfriend are making love. When does it happen: when I was out working. It is something that I hadn’t wanted to know. That it happened is already bad enough, but I hadn’t wanted to know. Sometimes I don’t care at all, sometimes I feel so betrayed, then I see myself again at Easter giving my boyfriend and sister an Easter egg and hearing myself say: “For the two people I love the most.” That my boyfriend cheated on me is not even the problem, but that my sister could so easily betray me I really cannot understand*
Maintaining social relations (*n* = 15)	*A good friend – that I like very much by the way – who has been a regular fellow passenger on the train. She often sat across from me and sometimes I see her in my mind, even though in reality no one is sitting across from me. A visualized memory of those travels, which do me good*
Warning signal (*n* = 12)	Memory*: I once had my toe stuck between something which made the nail almost fall off. The doctor then removed the nail and it looked horrible for a while. Every time I’m wearing thongs on the bicycle/walk behind a grocery cart I’m afraid that my toe will get stuck between. There is no special location/people present/event. Just the startle moment and the image of the bloody toe. It always frightens me and I’m scared it will happen again, it’s not that I’m afraid of the pain, but the way it looked just gives me the creeps. Then I don’t feel comfortable at all for a moment*
	Non-memory*: Someone is holding something valuable in their hand and I see in my mind how the objects falls down on the floor. Sometimes it’s just a bottle of wine held by my friend and I feel like I need to be vigilant and be ready to catch the bottle*
Mood repair (*n* = 8)	*In [city] at the quay during the [event]. Together with a colleague a meeting with a group of guys. It happened last year. We chat some and then party on for a long time all together. Evokes feelings of joy because it was a lot of fun and hoping to run into them again this year*.
Preparation (*n* = 5)	*It’s more thoughts than experiences. Because I study in [country] but am from [other country], I think about my parents a lot lately and thoughts of them come to mind. Partly I wonder what they are doing now and partly I think about what it would be like if they wouldn’t be there anymore, because they are near their 70s already. Then I do wonder whether I will be able to say that I have spent enough time with them. This is not necessarily negative for me. I’m thinking about them a lot and actively take on things, which makes me feel that we ARE spending an intensive amount of time together because I realize that the time together can end*

### Self-descriptions

The number of positive self-descriptions was significantly correlated with psychopathology, *r* = −0.31, *p* = 0.009, but was not associated with any of the other measures. The number of negative self-descriptions was significantly correlated with psychopathology, *r* = 0.43, *p* < 0.001, the number of negative involuntary cognitions, *r* = 0.32, *p* = 0.008, the number of sad involuntary cognitions, *r* = 0.36, *p* = 0.002, the level of sadness of these involuntary cognitions, *r* = 0.38, *p* = 0.001, the number of involuntary cognitions with guilt/shame content, *r* = 0.29, *p* = 0.016, and the number of involuntary cognitions that were assigned to the emotional processing function category, *r* = 0.38, *p* = 0.001.

### Psychopathology

SCL-90 scores were significantly correlated with the level of distress associated with cognitions that participants reported as distressing, *r* = 0.25, *p* = 0.037, number of cognitions that were associated with disgust, *r* = 0.29, *p* = 0.016, and the levels of disgust associated with those cognitions, *r* = 0.29, *p* = 0.015, number of cognitions in the form of bodily sensations, *r* = 0.322, *p* = 0.007, and the number of worst case scenario non-memory involuntary cognitions, *r* = 0.25, *p* = 0.037, and, as mentioned, the number of positive, *r* = −0.31, *p* = 0.009, and negative self-descriptions, *r* = 0.43, *p* < 0.001. Table 5 shows the Pearson correlations between each of the SCL-90 subscales (columns), and involuntary cognitions characteristics and self-descriptions (rows). Only variables with at least one significant correlation with the SCL-90 subscales are included.

**Table 5:** **Pearson correlations between the SCL-90 subscales, and characteristics of the involuntary cognitions and self-descriptions**.

	SOM	OBS	INT	DEP	ANX	HOS	PHO	PAR	PSY	ADD
**Emotion**
Distress	n.s.	n.s.	n.s.	n.s.	0.275*	0.240*	n.s.	n.s.	n.s.	n.s.
Disgust	n.s.	0.294*	0.308*	n.s.	n.s.	0.426***	0.330**	n.s.	n.s.	0.256*
Love	n.s.	n.s.	n.s.	n.s.	n.s.	n.s.	n.s.	n.s.	0.289*	n.s.
**Intensity**
Happiness	n.s.	n.s.	n.s.	n.s.	−0.258*	n.s.	n.s.	n.s.	n.s.	n.s.
Distress	0.242*	n.s.	n.s.	0.259*	0.316**	0.244*	n.s.	0.249*	n.s.	n.s.
Disgust	n.s.	0.301*	0.310*	n.s.	n.s.	0.409**	0.320**	n.s.	0.251*	0.255*
Love	n.s.	n.s.	n.s.	n.s.	n.s.	n.s.	n.s.	n.s.	0.289*	n.s.
**Quality**
Bodily sensation	0.349**	0.307*	0.282*	n.s.	0.385*	0.275*	0.532***	n.s.	n.s.	0.241*
**Type**
Worst case scenario	0.290*	n.s.	n.s.	n.s.	0.370**	0.340**	n.s.	n.s.	n.s.	n.s.
Rumination	n.s.	n.s.	n.s.	n.s.	n.s.	n.s.	n.s.	n.s.	0.316**	0.341**
**Content**
Life-threatening event	n.s.	0.246**	n.s.	n.s.	0.316**	n.s.	n.s.	n.s.	n.s.	n.s.
**Function**
Warning signal	n.s.	n.s.	n.s.	n.s.	0.318**	n.s.	n.s.	n.s.	n.s.	n.s.
**Self-descriptions**
Positive	n.s.	−0.362**	−0.358**	−0.236*	n.s.	−0.275*	−0.248*	n.s.	n.s.	n.s.
Negative	n.s.	0.427***	0.380**	0.436***	n.s.	0.350**	0.291*	0.357**	0.295*	0.269*

SOM, somatization; OBS, obsessive compulsive; INT, interpersonal sensitivity; DEP, depression; ANX, anxiety; HOS, hostility; PHO, phobic anxiety; PAR, paranoid ideation; PSY, psychoticism; ADD, additional items. *p < 0.05, **p < 0.01, ***p < 0.001.

### Comparisons between involuntary memories vs. involuntary non-memories

The finding that nearly half of the reported involuntary cognitions were not memories was of great interest to us, particularly in light of the surprising lack of empirical research into non-memory involuntary cognitions. The relative proportions of memories and non-memories therefore prompted us to consider the extent to which these two types of involuntary cognitions might overlap or differ in terms of their features, as well as the way in which they are subjectively experienced.

Chi-square analyses were conducted to compare memories and non-memories on valence (positive and negative), associated emotions, quality, content, and function. A *t*-test was conducted to compare the levels of emotion (i.e., emotional intensity) of the emotions associated with the involuntary cognitions. Memories vs. non-memories were equally often positive or negative, and there was no difference in the associated emotions or the emotionality (i.e., rated level of emotion) of the two types of cognitions, all *p*s >0.05.

There was a significant difference in the content of the involuntary cognitions between memories and non-memories, χ^2^(5) = 15.71, *p* = 0.008, and a difference in the function, χ^2^(4) = 10.51, *p* = 0.033. The difference in quality approached significance, χ^2^(4) = 9.19, *p* = 0.057. In regards to content, approximately half of all memory and non-memory involuntary cognitions were about relationships. After that, memories were most often about life-threatening events whereas non-memories were equally often about life-threatening events and achievement/mastery. That is, life-threatening events were twice as likely to be the content of memories as non-memories. Finally, none of the non-memories were about recreation/exploration, whereas about 21% of memories have this content.

In terms of possible function, memory involuntary cognitions were most often coded as having the possible function of emotional processing and social relations. Further, this function was assigned to memory involuntary cognitions twice as often as was the function of mood recovery or warning signals. Similarly, the function of emotional processing was most often assigned to non-memory involuntary cognitions. In contrast to memories, non-memories had the possible function of social relations, preparation, and warning signals equally often. Relatively speaking, the functions of emotional processing followed by social relations were assigned to memories and non-memories equally often. Memories tended to be associated with the possible function of mood repair relatively more often than non-memories. Non-memories tended to be associated with the possible functions of preparation and warning signal relatively more often than memories. In fact, none of the memories were coded as having the function of preparation.

The large majority of memories were predominantly visual in nature, whereas only about two-thirds of the non-memories were. This is perhaps not surprising because autobiographical memories by definition have a strong visual component whereas non-memories could also exist of purely verbal thoughts (e.g., ruminations).

## Discussion

The aim of this study was to explore the occurrence of involuntary cognitions in everyday life using a bottom-up approach. We use the term “involuntary cognitions” to refer to memories, thoughts, and mental images that pop up into mind spontaneously, without conscious effort to retrieve or produce them. To assess involuntary cognitions in everyday life, a PC administered structured interview was presented to an unselected group of 70 university students, most of them female. The interview queried participants about involuntary memories, thoughts, and images from the past month. By adopting a bottom-up approach, the exploratory analysis was more or less free of guidance from any specific theoretical framework, and was not limited to one specific type of cognition.

In this sample, 67.14% of participants reported at least one involuntary cognition in the past month. In terms of qualities or characteristics, all cognitions were considered to have at least some emotional impact, and the predominant emotions were “happy” and “sad.” In regard to sensory modality, 82.26% of the reported involuntary cognitions were rated as being predominantly visual in nature, which is in line with earlier research showing that the visual modality is often dominant in mental imagery [e.g., Ref. (24)]. Of all reported involuntary cognitions, only 61.29% were (autobiographical) memories, as indicated by the participants. This left us with 38.71% of “non-memories,” which were further categorized using available coding schemes and relevant descriptive literature (see Materials and Methods). The non-memories mainly consisted of daydreams, imagery of worst case scenarios, future events, hypothetical reconstructions of past events that were not solved for the individual (i.e., “missing pieces” in a coherent narrative), and verbal ruminations. These types of cognitions occurred more or less in equal numbers.

In terms of content, the three main categories that were observed were social relations (e.g., an image of a friend sitting across from you in the train), life-threatening events (e.g., a bloody accident), and recreation/exploration (e.g., relaxing on top of a hill with a great view). Possible or hypothetical functions were identified based on available literature (see Materials and Methods). Our examination led us to conclude that around 33% of the involuntary cognitions reported could be functional for emotional processing, 25% for maintaining social relationships, 20% as warning signals, 10% in mood repair, and another 10% in preparing for future events. Analyses that compared memories and non-memories revealed surprisingly few differences. Memories and non-memories were comparable in terms of valence, emotions, and emotional intensity. This may suggest that for the individual, the distinction between memories and non-memories may not in fact be important, as both types of involuntary cognitions represent fairly similar experiences, at least in terms of the characteristics that were measured in this study. Further, for the life-threatening events category, intrusive cognitions were twice as likely to be memories as opposed to non-memories, and there were no non-memories of the recreation/exploration categories. These findings may be methodological artifacts, because the inherent nature of the categories “life-threatening events” and “exploration/recreation” have a strong associations with personal past experiences.

For both memories and non-memories, the most prevalent potential function was emotional processing. Mood repair was more often associated with memories than non-memories, in line with previous evidence of the effectiveness of positive memory recall as a means by which to counter a sad mood state [e.g., Ref. (33)]. Prevalent potential functions for non-memories were warning signals and preparation for future events. For the potential function of preparation this is not surprising, as the cognition is about an event that has not happened yet and could therefore not be a memory [although it is likely that the information represented in the future oriented cognition heavily draws on information from past experiences; (41)]. This potential function of a “warning signal” was identified from the literature on psychological trauma, in which it has been proposed that involuntary autobiographical memories of negative events may occur in situations in which cues are present that could signal or predict a repeat of the past experience (22). In our study, we identified memories that could be interpreted as serving the function of a warning signal. For example, one participant had experienced her foot getting stuck in the wheel when cycling, which resulted in a bloody toe. She experienced an involuntary memory of this toe whenever the risk of a repeat of this accident was present (e.g., walking close behind a grocery trolley). The finding in our sample that it was not only memories served that function of a warning signal may suggest that non-memories may function as warning signals for negative experiences even if the individual has not experienced a similar experience him/herself before.

In addition to involuntary cognitions, self-descriptions and psychopathology were assessed using the Twenty-Statement Test (37), and the SCL-90 (38), respectively. Correlational analyses showed that whereas positive self-descriptions (e.g., “I am smart”) were negatively associated with psychopathology, they were not related to any characteristics of the involuntary cognitions. Negative self-descriptions (e.g., “I am ugly”), in contrast, were not only associated with levels of psychopathology, but also with the number of negative, mostly sadness-related involuntary cognitions, levels of sadness associated with the cognitions, the number of guilt/shame cognitions, and the hypothetical function of emotional processing. This latter relationship may indicate that emotional processing of one or more negative experiences could be a main goal for individuals with a negative self-image. This would be in line with the theoretical proposition that mental images are associated with an individual’s goals, which in turn are related to their self-image, according to the Self-Memory System (42). Higher scores on the SCL-90, indicating higher levels of psychopathology, were related to more distressing involuntary cognitions, and specifically higher numbers of disgust-related involuntary cognitions, greater levels of disgust, more involuntary cognitions involving bodily sensations, and more non-memories classified as worst case scenarios. Further, the SCL-90 subscales revealed many significant correlations with characteristics of involuntary cognitions and self-descriptions, supportive of a theoretical link between psychopathology, involuntary cognitions, and the self (21). Some general observations were that most correlations were positive (except for the correlation with intensity of happiness associated with happy involuntary cognitions), suggesting that the experience of involuntary cognitions is mostly associated with poorer mental health, and not with positive mental well-being. Further, some variables showed significant relations across multiple subscales of the SCL-90, such as intrusive bodily sensations, involuntary cognitions associated with distress or disgust, and the intensity of the associated distress or disgust, lower numbers of positive self-descriptions and higher number of negative self-descriptions. Although it is tempting to engage in theoretical interpretation of these correlations as some appear very intuitive (e.g., a correlation between involuntary cognitions about life-threatening events and the subscales Obsessive compulsive and Anxiety; and the function of warning signal and the Anxiety subscale) we should be extremely careful with any such interpretations as our findings await replication.

Because an unselected sample was recruited, it is possible and even likely that some participants suffered from psychological symptoms (whether subclinical, or consistent with a clinical diagnosis – e.g., obsessive compulsive disorder, panic disorder) at the time of testing, which may in part explain these findings. Further, the majority of participants in our sample were female and therefore the results may be more representative of females than males. Future research with samples of individuals who have been screened for the presence of psychopathological conditions will test this possibility.

This study has several limitations. First, the advantages of using a bottom-up approach also come with methodological disadvantages. The coding schemes that were used were based on existing literature where possible, but were not available or suitable for all our study goals. Therefore, we constructed several classifications on face value (e.g., “hypothetical reconstructions”). Overall inter-rater reliabilities for existing classifications varied from minimal (e.g., for quality coding) to moderate (e.g., type) to high (e.g., for content and self-descriptions). Therefore, the findings presented here are very preliminary and replication will be important. Notwithstanding these shortcomings, this approach allowed us to explore the phenomenon of involuntary cognitions in a way which resulted, in our opinion, in an intriguing pattern of findings. Further, the literature that was reviewed in the Section “Introduction” included different types of involuntary cognitions that have been identified in the cognitive and clinical literature. That is, involuntary autobiographical memories, involuntary semantic cognitions, and involuntary thoughts and images all come to mind unbidden, and in the case of cognitions of negative content or valence, are unwanted. However, our data showed that other mental phenomena, such as rumination and forms of mind wandering (e.g., daydreams) were sometimes similarly experienced as involuntary and spontaneous by our participants, even though in the literature rumination and daydreaming are typically not categorized as “intrusive” or “involuntary” cognitions (although their onset may be spontaneous). Thus, it may be warranted to broaden our view of the types of cognitions that can potentially be experienced as involuntary to include rumination and forms of mind wandering. Second, the structured interview assessed only three “main” involuntary cognitions that occurred during the month preceding the study. This is likely to be a gross underestimation of the number of involuntary cognitions that occur daily [c.f., (13, 17)], and frequency of occurrence of involuntary cognitions was not assessed which, although not the goal of this study, may be considered a limitation as cognitions with a high frequency of occurrence may also differ from low frequency cognitions. In addition, we did not assess triggers, which could have provided further information about the content and function of the involuntary cognitions.

Relatedly, the involuntary cognitions that were reported all had some emotional impact (with means ranging from *M* = 4.83 to *M* = 6.33 on a scale from 1 to 7), which is likely at least partly due to a retrospective bias, such that more significant or emotional cognitions will be remembered whereas less significant or emotional cognitions, which are perhaps also more frequent, may be forgotten. Nevertheless, emotional cognitions may be more informative for some purposes as these are more likely to translate to behavior [e.g., actually preparing for a future event, avoiding a negative consequence, etc., (34)]. Third, all cognitions that were reported were classified on all factors of interest (quality, type, content, and function), resulting in overlapping categories. For example, a cognition with a function of maintaining social relations most likely overlaps with cognitions with a content of social relations, and cognitions for mood repair and cognitions about recreation/exploration likely overlap because of their positive valence. In light of this, the findings therefore need to be interpreted with caution. Finally, although the findings of this study show that there are many different types of involuntary cognitions, each with its own specific quality and characteristics and potential function, the nature of this study prevents us from drawing firm conclusions about the relations between these characteristics. Prospective and experimental studies are needed to test their empirical significance.

To summarize, we present several interesting findings that provide food for thought for both clinical and cognitive psychologists who are interested in involuntary cognitions. The main goal of this study was to explore the quality, type, content, and function of involuntary cognitions in everyday life, without limiting focus to a specific subtype (e.g., autobiographical memories, positive or negative cognitions). One of the main findings was that involuntary memories differ very little from other involuntary cognitions on important markers such as valence and emotionality. This raises the question as to whether the distinction between memories and non-memories is actually an important one in the experience of the individual. Interestingly, specific relationships emerged between psychopathology and negative self-descriptions, as well as the characteristics of cognitions. In our view, these are exciting preliminary findings that we hope may spark future research into involuntary cognitions and their specific impact on the individual’s experience and behavior.

## Conflict of Interest Statement

The authors declare that the research was conducted in the absence of any commercial or financial relationships that could be construed as a potential conflict of interest.
